# Risk factors for postoperative cerebral infarction in Lung Cancer patients: a retrospective study

**DOI:** 10.1186/s13019-023-02220-6

**Published:** 2023-04-11

**Authors:** Natsumi Maru, Haruaki Hino, Takahiro Utsumi, Hiroshi Matsui, Yohei Taniguchi, Tomohito Saito, Tomohiro Murakawa

**Affiliations:** Department of Thoracic Surgery, Kansai Medical University, 2-3-1 Shinmachi Hirakata-shi, Osaka, Osaka 573-1191 Japan

**Keywords:** Cerebral infarction, Lung cancer surgery, Vessel stapling, Thrombus formation

## Abstract

**Background:**

Postoperative cerebral infarction is a rare but serious complication after lung cancer surgery. We aimed to investigate the risk factors and evaluate the efficiency of our devised surgical procedure to prevent cerebral infarction.

**Methods:**

We retrospectively examined 1,189 patients who underwent a single lobectomy for lung cancer at our institution. We identified the risk factors for cerebral infarction and investigated the preventive effects of performing resection of the pulmonary vein as the last step of the surgical procedure during left upper lobectomy.

**Results:**

Among the 1,189 patients, we identified 5 male patients (0.4%) with postoperative cerebral infarction. All five underwent left-sided lobectomy including three upper and two lower lobectomies. Left-sided lobectomy, a lower forced expiratory volume in 1 s, and lower body mass index were associated with postoperative cerebral infarction (*P*s < 0.05). The 274 patients who underwent left upper lobectomy were stratified by two procedures: lobectomy with resection of the pulmonary vein as the last step of the surgical procedure (n = 120) and the standard procedure (n = 154). The former procedure significantly shortened the length of the pulmonary vein stump when compared with the standard procedure (mean stump length: 15.1 vs. 18.6 mm, *P* < 0.01), and the shorter pulmonary vein might possibly prevent postoperative cerebral infarction (frequency: 0.8% vs. 1.3%, Odds ratio: 0.19, P = 0.31).

**Conclusions:**

Resecting the pulmonary vein as the last step during the left upper lobectomy enabled the length of the pulmonary stump to be significantly shorter, which may contribute to preventing cerebral infarction.

**Supplementary Information:**

The online version contains supplementary material available at 10.1186/s13019-023-02220-6.

## Background

Postoperative cerebral infarction (CI) is a rare but severe complication, occurring in 0.6–1.6% of patients undergoing surgery for lung cancer. The highest incidence was reported to be approximately 4%, especially in left upper lobectomy (LUL) [1–5]. Recent studies have shown that thrombus formation at a relatively long stump of the pulmonary vein (PV) following LUL is a risk factor for postoperative CI [6–9]. Some studies have also shown that thrombus formation at the PV stump, which is considered a cause of postoperative CI, can be prevented by shortening the length of the PV stump [10–13]. However, no study has shown resecting the PV at the nearest left atrium during LUL as a last step of the surgical procedure using an endo-stapler, to shorten the length of the PV stump. Therefore, we aimed to elucidate the risk factors for postoperative CI and investigate the impact of our devised procedure in patients undergoing lung cancer surgery.

## Materials and methods

### Patients

We conducted a retrospective study by reviewing the medical records of patients with lung cancer who underwent surgery at Kansai Medical University Hospital, Osaka, Japan, between January 2006 and December 2020. The inclusion criteria were as follows: lobectomy, complete resection, and non-small-cell lung cancer. The exclusion criteria were as follows: bilobectomy, pneumonectomy, preoperative chemo- and/or radiotherapy, synchronous multiple lung cancer, incomplete resection (R1 and 2), and histological features including small cell carcinoma and adenocarcinoma in situ. We gathered clinical data including age, sex, body mass index (BMI), pulmonary function including vital capacity (VC), percentage of forced expiratory volume in 1 s/ forced vital capacity (FEV_1_/FVC), smoking history (pack per year), preoperative carcinoembryonic antigen (CEA) level, comorbidities including atrial fibrillation (Af) and diabetes mellitus (DM), history of cancer within 5 years and CI, perioperative anticoagulant therapy, operative procedure, operation time, bleeding amount, postoperative Af, postoperative stay, clinical and pathological stages, and histological findings. We measured the length of the PV stump using horizontally enhanced or non-enhanced computed tomography (CT) images of the patients after LUL. We also collected survival data after lung cancer surgery. Overall survival (OS) was calculated as the time from the date of surgery to the date of death or the last follow-up. Recurrence-free survival (RFS) was calculated from the date of surgery to the date of recurrence. We defined postoperative CI as occurring within 90 days after surgery. CI was diagnosed by neurologists at our institution considering both physical symptoms and magnetic resonance imaging findings at the onset of CI. Tumor stage was determined according to the eighth edition of the TNM staging system of the International Union against Cancer [14]. The histological tumor type was determined according to the 2015 World Health Organization Classification of Tumors [15]. Ethical approval for this study was granted by the Ethics committee of Kansai Medical University Hospital (approval number: 2021214, dated November 16, 2021).

### Surgical procedure

**Fig. 1:**
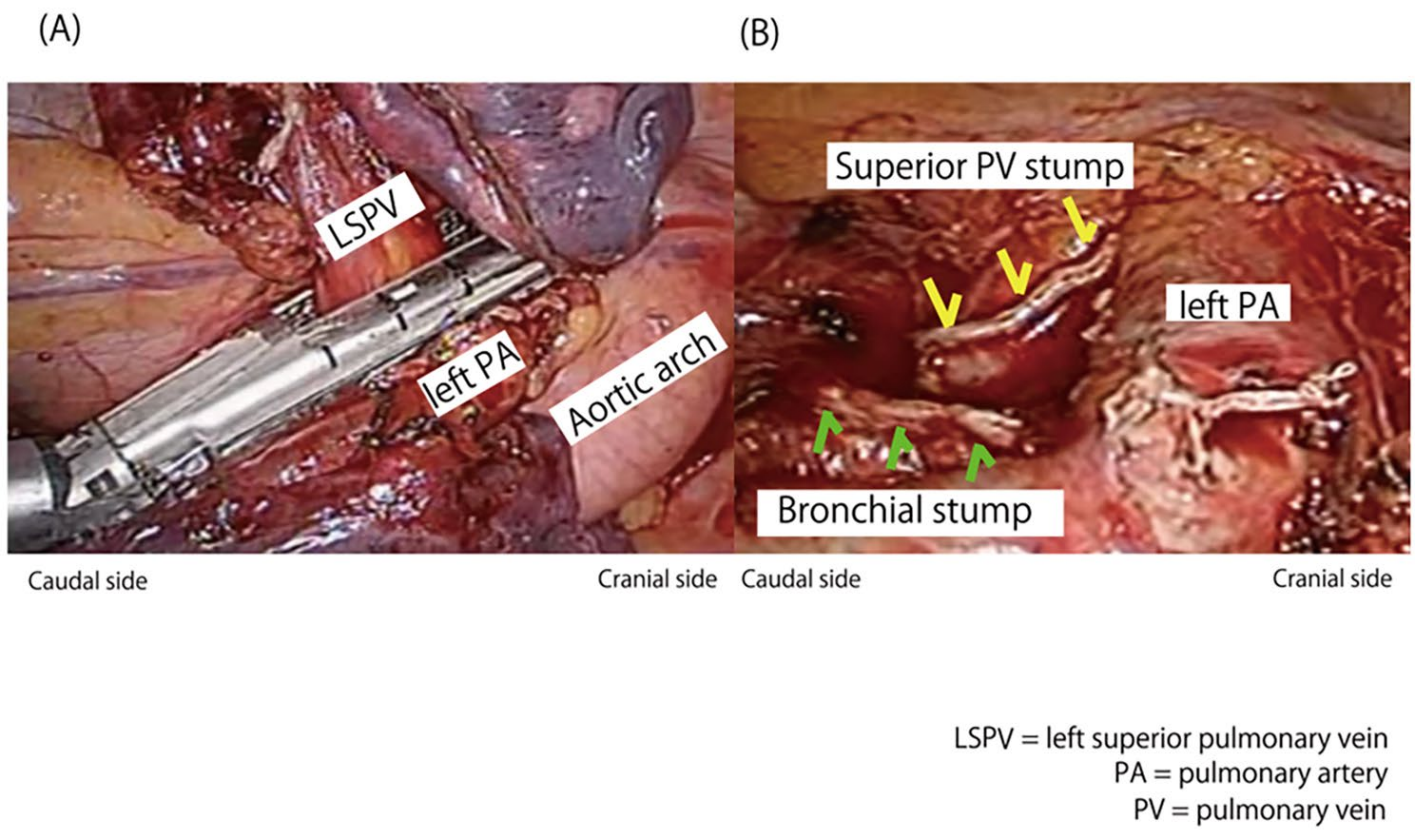
Operative view of the pulmonary vein stump in the left thoracic cavity **(A)** Surgical view during stapling of a pulmonary vein as a last step of the surgical procedure. **(B)** Surgical view after dissecting the pulmonary vein as a last step of the surgical procedure LSPV, left superior pulmonary vein; PA, pulmonary artery; PV, pulmonary vein

All patients underwent anatomical lobectomy using automated suture devices to dissect the PV. Considering the evidence that thrombus formation at the PV stump might cause postoperative CI after LUL, we intentionally changed the order of dissection of the pulmonary vessels during LUL starting May 2016. We dissected the PV as a last step of the surgical procedure, after resecting the left upper bronchus, pulmonary artery, and parenchyma of the interlobar fissure. We aimed to shorten the residual PV stump by lifting the left upper lobe with counterattraction and dissecting the PV as close to the root of the left atrium as possible, without opening the pericardium (Fig. 1). We named the method “PV-last procedure’’. We performed the PV-last procedure via video-assisted thoracoscopic surgery as the first choice, regardless of cancer staging, extent of interlobar fissure, and cancer size and location. However, we performed a standard procedure (non-PV-last procedure, dissecting the PV before pulmonary artery resection) for complicated cases, such as those involving lymph node metastasis or bleeding accidents. We divided the patients who underwent LUL into two groups: those who received PV-last procedure and those who received non-PV-last procedure, to analyze the impact of the surgical procedure on postoperative outcomes. When we resected left superior pulmonary vein (LSPV), we usually used Endo GIA ^TM^ Universal Staplers and Tri-StapleTM 30mm or 45mm, curved chip Gray (Medtronic, Dublin, Ireland).

### Statistics

Fisher’s exact test was used to compare the categorical variables. When we compared the continuous variables, we used the Mann–Whitney U test for all cohorts divided by the presence of CI, and Student’s t-tests for cohorts with LUL divided by the two procedures. We performed univariate and multivariate logistic regression analyses to assess the risk factors associated with CI and the preventive effect of the PV-last procedure on CI complications. All variables showed significance in the nominal two-tailed test (P < 0.1), and postoperative risk factors for CI, such as age, sex, DM, and LUL, were entered into a binary logistic regression model. When we measured PV stump length, there was a concern that the time to CT imaging could affect the length of the PV stump. Therefore, propensity score matching was performed to align the median time to CT imaging between patients with PV-last and non-PV-last procedures. In addition, we analyzed and compared the OS and RFS rates in each group, divided by PV-last and non-PV-last procedures, using the Kaplan–Meier method. We used EZR (Saitama Medical Center, Jichi Medical University, Saitama, Japan), a graphical user interface for R (The R Foundation for Statistical Computing, Vienna, Austria), to compare patients’ clinical characteristics [16] and the JMP software (ver. 12; SAS Institute Inc., Cary, NC, USA) for survival and logistic regression analyses. Statistical significance was set at P value < 0.05.

## Results

There were 1,803 lung cancer surgeries performed from 2006 to 2020 at our institute, and a total of 1,189 patients were included in this study. CI was observed in five patients (0.42%), three of whom had undergone LUL and two of whom had undergone left lower lobectomies. The clinical characteristics of 1,189 patients (741 right-side lobectomies and 449 left-side lobectomies) are presented in Table 1. Postoperative CI was significantly associated with left-sided lobectomy and a lower FEV_1_/FVC ratio and BMI (*P*s < 0.05). However, other clinicopathological features, including age, sex, comorbidities, and cancer stage, were not significantly associated with postoperative CI. The characteristics of the five patients who had postoperative CI are shown in Table 2. CI onset was mostly in the early phase after lobectomy (mean 4.5 days, median 1, and range 1–13), and no one experienced postoperative Af. An enhanced CT was performed for all five patients after the onset of CI; however, thrombus formation at the PV stump was not detected in anyone. After the clinical course of CI, four patients were discharged and one was transferred to another hospital for rehabilitation. The results of the univariate and multivariate analyses of the risk factors associated with CI are shown in Table 3. Lower BMI and FEV_1_/FVC were marginal risk factors among pre- and perioperative variables (*P* = 0.06 and 0.074, respectively); however, LUL was not (*P* = 0.99).

**Table 1:** Clinical characteristics of all 1,189 patients undergoing single lobectomy

	Cerebral infarction (+) (n = 5)	Cerebral infarction (-) (n = 1184)	*P* value
Age (years), median (IQR)	68 (67–71)	71 (64–76)	0.67
Male, n (%)	5 (100)	707 (60.0)	0.09
**Body mass index, median (IQR)**	**19.9 (19.5–20.3)**	**22.6 (20.6–24.6)**	0.026*
Percentage of VC, median (IQR)	98.9 (89.1–91.5)	90.3 (89.3–110.7)	0.067
**FEV1.0%, median (IQR)**	**59 (58.1–70.2)**	**74.2 (67.6–79.7)**	0.044*
Pack per year ≤ 600, n (%)	2 (40.0)	645 (54.5)	0.66
> 600, n (%)	3 (60.0)	492 (41.5)	
Unknown, n (%)	0 (0)	47 (4.0)	
CEA ≤ 5, n (%)	2 (40.0)	743 (62.7)	0.33
> 5, n (%)	2 (40.0)	298 (25.2)	
Unknown, n (%)	1 (20.0)	143 (12.1)	
Af, n (%)	0 (0)	49 (4.1)	1
Diabetes mellitus, n (%)	1 (20.0)	165 (14)	0.53
History of cerebral infarction, n (%)	1 (20.0)	116 (9.7)	0.41
History of cancer within 5 years, n (%)	1 (20.0)	200 (17.0)	1
Anticoagulant therapy, n (%)	1 (20.0)	117 (9.8)	0.41
Charlson Comorbidity Index 0 or 1, n (%)	4 (80.0)	901 (76.0)	1
2, n (%)	1 (20.0)	283 (24.0)	
Procedure RUL, n (%)	0	410 (34.6)	0.051
RML	0	79 (6.7)	
RLL	0	252 (21.3)	
LUL	3 (60.0)	271 (22.9)	
LLL	2 (40.0)	173 (14.6)	
**Left-side lobectomy**	**5 (100.0)**	**444 (37.4)**	0.0075*
Operation time (min), median (IQR)	106 (93–166)	151 (111–228)	0.15
Bleeding amount (mL), median (IQR)	70 (5–85)	57.5 (18.5–121.5)	0.56
Postoperative atrial fibrillation, n (%)	0	18 (1.5)	1
Postoperative stay, mean (IQR)	11 (8–36)	11 (8–14)	0.77
Clinical stage I, n (%)	4 (80.0)	984 (83.1)	0.61
II, n (%)	1 (20.0)	154 (13.0)	
III, n (%)	0	44 (3.7)	
Pathological stage I, n (%)	4 (80.0)	827 (69.8)	0.64
II, n (%)	0	198 (16.7)	
III, n (%)	1 (20.0)	159 (13.4)	
Histology of adenocarcinoma, n (%)	3 (60.0)	845 (71.4)	0.56
Squamous cell carcinoma, n (%)	2 (40.0)	234 (19.8)	
Others, n (%)	0	105 (8.9)	
Observation time, months, median (IQR)	29.6 (24.3–44.5)	30.7 (11.7–61.3)	0.94

VC, vital capacity; FEV1.0%, percentage of forced expiratory volume in 1 s; FVC, forced vital capacity; CEA, carcinoembryonic antigen; Af, atrial fibrillation; RUL, right upper lobectomy; RML, right middle lobectomy; RLL, right lower lobectomy; LUL, left upper lobectomy; LLL, left lower lobectomy; IQR, interquartile range; min, minutes

**Table 2:** Clinical characteristics of five patients who presented with postoperative cerebral infarction

Case	Age	Gender	BMI	FEV1 /FVC	Pack per year	Preoperative comorbidity	Surgical procedure	Operation time (min)	Postoperative CI onset date	Pathology	Stage
1	67	M	19.5	59	0	Hypertension	LUL non-PV-last	166	3	Adenocarcinoma	IB
2	68	M	21.7	78.3	360	None	LUL non-PV-last	93	1	Adenocarcinoma	IA1
3	71	M	20.3	58.1	1000	DM, CI, Hypertension	LUL PV-last	87	13	Squamous cell carcinoma	IA2
4	75	M	19.9	48	1140	Tongue cancer	LLL	169	1	Adenocarcinoma	IB
5	67	M	18.2	70.2	1200	None	LLL	106	1	Squamous cell carcinoma	IIIB

DM, diabetes mellitus; LUL, left upper lobectomy; LLL, left lower lobectomy; FEV1, forced expiratory volume in 1 s; FVC, forced vital capacity; BMI, body mass index; PV, pulmonary vein; PV-last, procedure of dissecting pulmonary vein last; non-PV-last, a procedure of not dissecting pulmonary vein last; CI, cerebral infarction

**Table 3:** Analyses of risk factors for postoperative cerebral infarction of 1189 patients undergoing single lobectomy

	Univariate analysis			Multivariate analysis		
	Odds ratio	95% CI	*P* value	Odds ratio	95% CI	*P* value
Age (years)	0.99	0.908–1.1	1	0.96	0.845–1.09	0.54
Male / Female	4.47E + 07	0–Inf	1	7.89E + 07	0–Inf	0.99
Percentage of VC	0.956	0.903–1.01	0.12	0.976	0.914–1.04	0.45
FEV_1_/FVC	0.925	0.867–0.988	0.02	0.939	0.876–1.01	0.074
Pack per year > 600 / ≤ 600	1.07	0.32–11.8	0.46			
Body mass index	0.742	0.543–1.01	0.06	0.705	0.49–1.01	0.06
CEA > 5 / ≤ 5	2.5	0.35–17.8	0.36			
Af (yes / no)	7.20E-07	0–Inf	0.99			
DM (yes / no)	1.5	0.17–13.9	0.7	1.94	0.156–24.1	0.61
History of cerebral infarction (yes / no)	2.3	0.26–20.8	0.46			
History of cancer within 5 years (yes / no)	1.23	0.13–11.1	0.85			
Anticoagulant therapy (yes / no)	2.3	0.253–20.6	0.46			
Charlson Comorbidity Index ≥ 2 / 0 or 1	0.8	0.08–7.15	0.84			
Procedures						
LUL/others	5.05	0.84–30.4	0.077	1.01	0.15–6.82	0.99
Left side / Right side	7.13E + 07	0–Inf	0.99	1.45E + 08	0–Inf	0.99
Postoperative Af (yes/no)	2.02E-06	0–Inf	1			
Clinical stage I	1	1				
II	1.66	0.185–15.0	0.65			
III	7.87E-08	0–Inf	0.99			
Pathological stage I	1	1				
II	8.92E-08	0–Inf	0.99			
III	1.31	0.146–11.8	0.81			
Histology Adenocarcinoma	1	1				
Squamous cell carcinoma	2.42	0.4–14.5	0.34			
Others	1.21E-07	0–Inf	0.99			

VC, vital capacity; FEV_1_, forced expiratory volume in one second, FVC forced vital capacity; Af, atrial fibrillation; DM, diabetes mellitus; CEA, carcinoembryonic antigen; LUL, left upper lobectomy; CI, confidence interval; inf, infinity

**Fig. 2:**
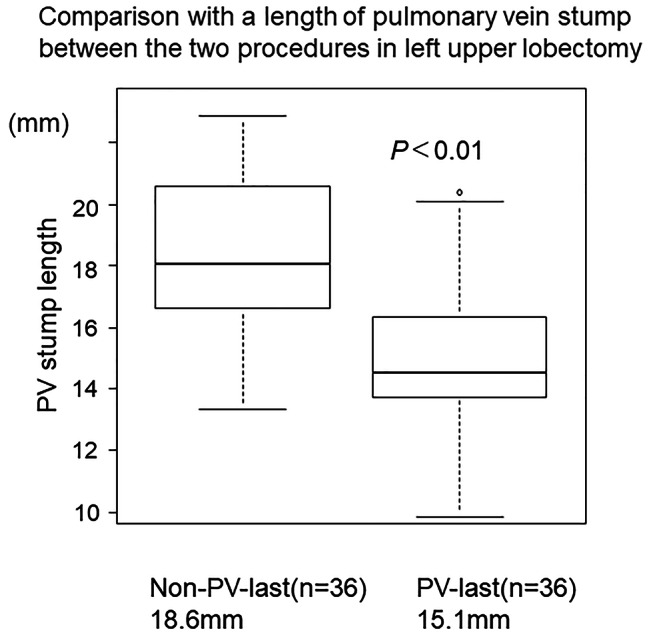
Box plot showing the length of the resected pulmonary vein after different lobectomy procedures The length of the pulmonary vein (PV) stump after the PV-last procedure was significantly shorter than that after the non-PV-last procedure (*P* < 0.01)

Among the 274 lung cancer patients who underwent LUL, we performed the PV-last procedure in 120 and the non-PV-last procedure in 154. The clinicopathological characteristics of patients in each group are shown in Additional Table 1. Patients who underwent the PV-last procedure had a significantly lower respiratory function, a shorter operation time, fewer bleeding events, and a shorter postoperative stay (*P*s < 0.05); however, the rates of all postoperative complications were not significantly different (*P* = 0.28). We measured the lengths of the PV stumps among propensity score mathced patients with the PV-last (n = 36) and the non-PV-last (n = 36) procedures who underwent CT after surgery (Fig. 2). The median time to CT imaging in the PV-last procedure was 515.5 days; it was 612.5 days in the non-PV-last procedure (P = 0.78) The mean length of the PV stump in the PV-last procedure (15.1 mm) was significantly shorter than that in the non-PV-last procedure (18.6 mm) (*P* < 0.01). Postoperative CI was observed in one patient who underwent the PV-last procedure (1/120, 0.8%) and two patients in the non-PV-last procedure (2/154, 1.3%), which did not show any significant difference (*P* = 1). The results of the univariate and multivariate analyses to evaluate the preventive effect of the PV-last procedure on CI are shown in Table 4. The PV-last procedure had a slightly favorable effect in preventing CI, when compared with the non-PV-last procedure; however, a significant difference was not observed (odds ratio, 0.19; *P* = 0.31).

**Table 4:** Analyses of risk factors for postoperative cerebral infarction of 274 patients undergoing LUL.

	Univariate analysis			Multivariate analysis		
	Odds ratio	95% CI	*P* value	Odds ratio	95% CI	*P* value
Age (years)	0.994	0.88–1.12	0.92	0.97	0.832–1.13	0.7
Male / Female	4.36E + 07	0–Inf	1	1.56E + 08	0–Inf	0.99
Percentage of VC	0.948	0.882–1.02	0.14	0.954	0.874–1.04	0.3
FEV1.0%	0.939	0.86–1.03	0.16	0.939	0.839–1.05	0.27
Pack per year > 600 / ≤ 600	0.66	0.059–7.4	0.74			
Body mass index	0.77	0.51–1.15	0.2	0.664	0.382–1.15	0.15
CEA > 5 / ≤ 5	5.14	0.46–57.6	0.19			
Af (yes / no)	8.E-07	0–Inf	1			
DM (yes / no)	3.37	0.29–38.2	0.33	3.96	0.19–82.3	0.37
Histology of cerebral infarction (yes / no)	3.E-07	0–23.6	1			
History of cancer within 5 years (yes / no)	8.85E-08	0–Inf	1			
Anticoagulant therapy (yes / no)	7.47	0.65–86.6	0.11			
Charlson Comorbidity Index 2 / 0 or 1	0	0–7.81	1			
Operation time (min)	0.98	0.948–1.01	0.24			
Bleeding amount (mL)	0.998	0.987–1.01	0.77			
Procedures PV-last / non-PV-last	0.64	0.057–7.13	0.72	0.191	0.00799–4.55	0.31
Clinical stage I	1	1				
II	0	0–18.75	1			
III	0	0–90.7	1			
Pathological stage I	1	1				
II	0	0–10.2	1			
III	0	1–13.1	1			
Histology Adenocarcinoma	1	1				
Squamous cell carcinoma	2.075	0.035–40.6	0.48			
Others	0	0–47.5	1			

VC, vital capacity; FEV1.0%, percentage of forced expiratory volume in one second; Af, atrial fibrillation; DM, diabetes mellitus; CEA, carcinoembryonic antigen; CI, confidence interval; LUL, left upper lobectomy; PV pulmonary vein

**Fig. 3:**
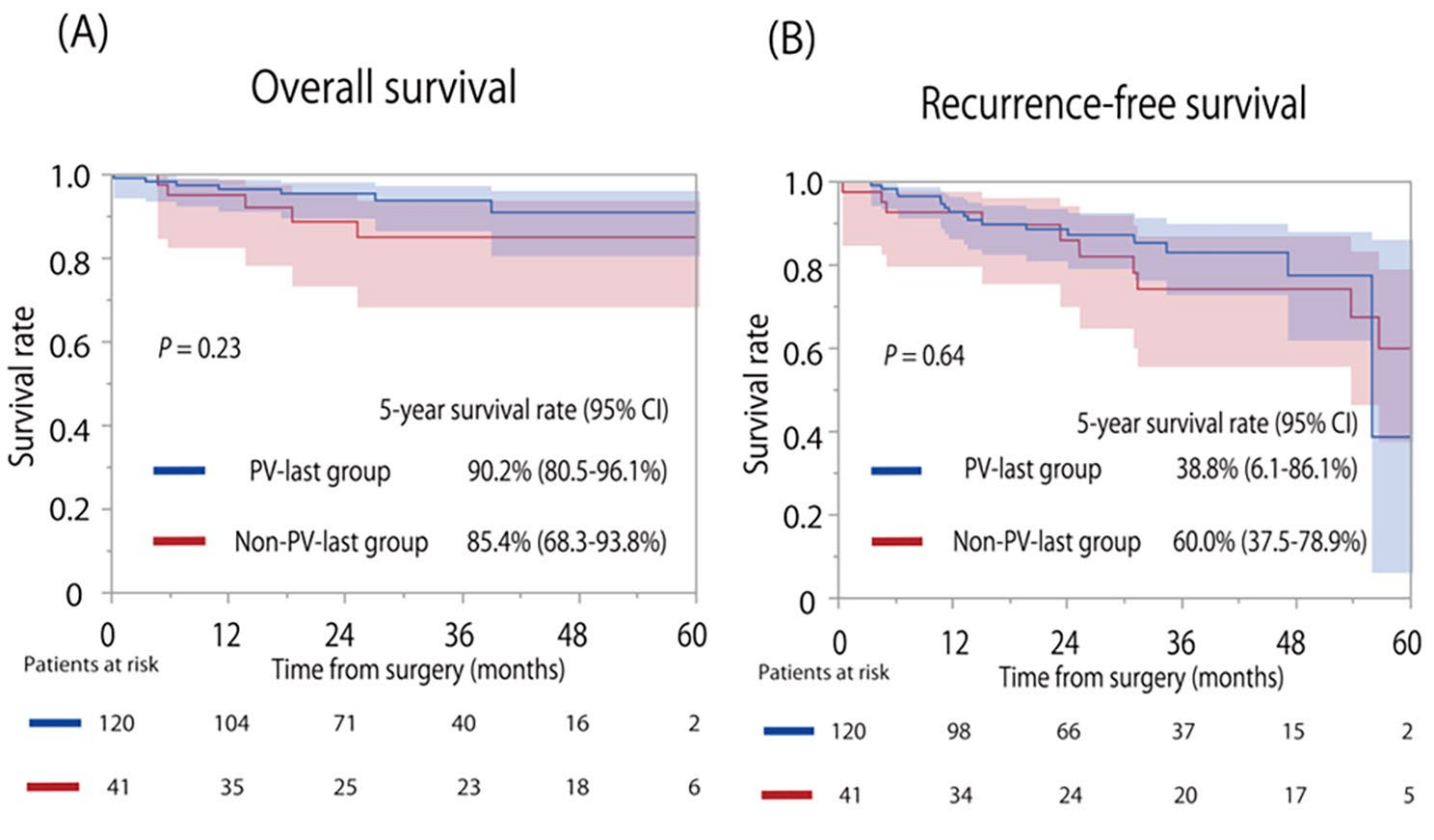
Kaplan–Meier survival curves between the PV-last and non-PV-last procedures **(A)** Overall survival curves; **(B)** Recurrence-free survival curves PV, Pulmonary vein

Survival curves for OS and RFS are shown in Fig. 3A and B. There were no significant differences between the two groups (P = 0.23 and 0.64, respectively). The median follow-up period after surgery was 25.9 months in patients with the PV-last procedure, and 37.4 months in patients with the non-PV-last procedure. The 5-year OS rates of the PV-last and non-PV-last procedures were 90.2% (95% confidence interval: 80.5–96.1%) and 85.4% (95% confidence interval: 68.3–93.8%), respectively, and the 5-year RFS rates were 38.8% (95% confidence interval: 6.1–86.1%) and 60.0% (95% confidence interval: 37.5–78.9%), respectively.

## Discussion

The incidence of postoperative CI after lobectomy was 0.4% (5/1189). Left-sided lobectomy, lower values of FEV_1_/FVC, and a lower BMI were significantly associated with CI in our single-institute retrospective study. In previous publications, the incidence of postoperative CI was 0.6–1.6% and LUL was a significant risk factor for postoperative CI [1–5, 12, 17], which was almost consistent with our results. Other risk factors, such as older age, male sex, and DM have also been reported [17, 18]. Lower values of FEV_1_/FVC could have been related to chronic obstructive pulmonary disease (COPD). It is known that COPD, which presents a decrease in the FEV_1_/FVC as a symptom, leads to increased complications of cardiovascular disease. Repeated hypoxic exposure causes vascular remodeling, which leads to the formation of unstable plaques, the rupture of which may result in CI. It has also been reported that the severity of COPD correlates with the degree of systemic inflammation, followed by arterial stiffness. These steps may be related to the development of CI. [19]. Several epidemiological studies in Japan have reported an increased risk of CI in men with low BMI. Although the association between physiological factors and proneness to CI remains unknown, low BMI may result in health risks such as insufficient nutrition and physical fitness, inflammation, and an unstable hormonal milieu [20]. We also considered the possibility of the presence of confounding factors that were not evaluated in this study.

A recent review summarized the causes of CI after lobectomy [8]. There are two pathways for thrombus formation: (I) postoperative paroxysmal Af, or blood flow change in the left atrium, and (II) a redundant PV stump. Hypercoagulability induced by lung cancer may promote thrombus formation through these pathways. Concerning the pulmonary vein stump, previous studies showed that the length of the PV stump after LUL was relatively longer than that in other types of lobectomies considering anatomical features; the root of the LSPV has a longer running length before penetrating the pericardium than other pulmonary veins of the right upper and lower limbs [6, 7, 9]. The average length of the LSPV after lobectomy was approximately 2.0 cm, whereas lengths of the other pulmonary veins ranged from 0.5 to 0.8 cm [21]. Owing to a long PV stump neighboring the left atrium, turbulent flow or stasis of blood likely occurs when the left PV flow is closed after lobectomy. Additionally, thrombus-promoting flow patterns, such as multidirectional flow and ascending flow in the PV stump after LUL, were observed on four-dimensional-flow magnetic resonance imaging, which supports our explanation [10, 22]. In summary, a turbulent flow change in the left atrium, sometimes complicated by Af, and/or an anatomical difference in the prolonged root of the left pulmonary vein were considered the main causes of postoperative CI after lung surgery, especially in LUL, considering prior publications and our results.

Recently, it was reported that proximal ligation of the PV to shorten the length of the PV stump was performed to prevent postoperative CI after LUL with potential efficacy [10–13]. The proximal ligation method at the intrapericardium was reported to shorten the length of the PV stump by 3 to 10 mm, when compared with the common procedure of dissecting the PV outside the pericardium using an endo-stapler [10, 11]. Proximal ligation results in a round residual PV stump and avoids vascular wall injury, contrary to the endo-stapler [13]. Even this method creates a longer PV stump after LUL than after other types of lobectomies owing to anatomical differences. However, most of these studies reported a relatively positive effect on preventing thrombosis at the PV stump and postoperative CI. Considering this, we attempted to resect the PV close to the orifice of the left atrium to shorten the length of the PV stump using an endo-stapler with countertraction, enabling us to safely perform the vascular approach without opening the pericardium (Fig. 1). The length of the PV stump in the PV-last procedure was significantly shorter than that in the non-PV-last procedure, supporting its validity (Fig. 2). Furthermore, the PV-last procedure achieved almost the same PV stump length as previously reported using the ligation method. Considering the frequency of CI, the incidence of CI in patients with the PV-last procedure (0.8%) was significantly lower than that previously reported (0.93–4.7%), which suggests that the PV-last procedure might help prevent postoperative CI [1–5, 12].

According to past publications, there has not been any recommended length of PV stump to prevent postoperative CI thus far. Procedures that excessively shorten the PV stump may lead to unexpected complications, such as cardiac tamponade [23]. Specifically, although the length of the PV stump by the PV-last procedure was not likely to be as short as that of other lobes, we considered that the length by our procedure was acceptable based on the evidence that the number of CI was lower and there were no obvious complications. As for the shape of the PV stump, compared with the proximal ligation method, the PV-last procedure makes the PV stump square by using a linear stapler, which could have a higher possibility of forming thrombus at the PV stump than the proximal ligation method. However, because there were several reported etiologies for postoperative CI, we assumed that only the shape of the stump did not have a high value for the complication. Despite the various etiologies for postoperative CI, procedures shortening the length of the PV stump for LUL contributed to preventing thrombus formation and postoperative CI, regardless of how the left upper PV was resected (ligating at the intrapericardium or stapling at the pericardium with countertraction). A large cohort study might clarify the correlation between PV stump length and the risk of CI.

There is evidence that dissecting a PV during lobectomy reduces tumor cell dissemination in the blood stream, preventing “circulation tumor cells [23–26]”. An improved survival outcome was obtained in lung cancer patients who first underwent PV resection, when compared with those who underwent pulmonary artery resection. In contrast, other studies showed that survival time was unaffected by the order in which the pulmonary vessels were dissected [27, 28]. We investigated if survival time differed between the PV-last and non-PV-last procedures. There was no difference between the two in OS and RFS (Fig. 3). During surgery we avoided manipulating cancerous lobes as much as possible to prevent circulating tumor cells, which may have contributed to the survival equivalence of the PV-last procedure. Future LUL performed with the PV-last procedure may support our results.

Our study has some limitations. First, we performed a retrospective study in a single institution, and the frequency of CI was relatively rare. Second, we did not perform enhanced CT for patients who underwent LUL. Therefore, we could not evaluate the presence of thrombus in the PV stump in the early phase. Third, there is the possibility of biases and restrictions affecting the PV-last procedure on a case-by-case basis. However, no increased morbidities related to the procedure were observed, with equivalent survival times in our study. We also demonstrated the technical efficacy and safety of the PV last procedure, which allowed us to definitively resect the left superior pulmonary vein in the case of the common pulmonary vein truncus, which branches into superior and inferior pulmonary veins outside of the pericardium. Therefore, despite its limitations, the PV-last procedure might be an acceptable procedure for LUL to prevent postoperative CI.

## Conclusion

Left-sided lobectomy, lower FEV_1_/FVC values, and a lower BMI were significantly associated with postoperative CI. Possibly, the PV-last procedure might reduce the risk of postoperative CI by shortening the length of the PV stump. A large cohort of multicenter studies should be conducted to confirm these findings and the potential of our procedure for general use in the planning of lobectomies, especially in LUL.

## Electronic supplementary material

Below is the link to the electronic supplementary material.

**Supplementary Material 1: Additional Table 1**

## Data Availability

The data that support the findings of this study are available on request from the corresponding author.

## Abbreviations

CI: Cerebral infarction
PV: Pulmonary vein
LUL: Left upper lobectomy
CEA: Carcinoembryonic antigen
CT: Computed tomography
DM: Diabetes mellitus
Af: Atrial fibrillation
OS: Overall survival
VC: Vital capacity
FEV_1_: Forced expiratory volume in one second
FVC: Forced vital capacity
COPD: Chronic obstructive pulmonary disease
LSPV: Left superior pulmonary vein
