# Supplementary material for: Risk factors for postoperative cerebral infarction in Lung Cancer patients: a retrospective study
Source: J Cardiothorac Surg. 2023 Apr 11;18:132. doi: 10.1186/s13019-023-02220-6 (PMC10091688; doi:10.1186/s13019-023-02220-6)
Supplement: Supplementary file 1 — Supplementary Material 1: Additional Table 1 [file 13019_2023_2220_MOESM1_ESM.docx]

**Additional Table 1. Clinical characteristics in the PV-last and non-PV-last groups.**

|  | PV-last procedure (n=120) | Non-PV-last procedure (n=154) | *P* value |
| --- | --- | --- | --- |
| Age, mean (range) | 70.4 (38~84) | 68.3 (38~84) | 0.057 |
| Male, n (%) | 63 (52.5) | 100 (65) | 0.047 |
| **Percent of VC, mean (range)** | **94.6 (54.7~143.6)** | **103.7 (58.1~151.5)** | **<0.001** |
| **FEV1.0%, mean (range)** | **71.3 (30.6~96.3)** | **73.9 (41.2~87.4)** | **0.019** |
| Pack-per-year ≤ 600, n (%) | 72 (60) | 77 (50%) | 0.26 |
| >600, n (%) | 46 (38.3) | 66 (43) |  |
| unknown, n (%) | 2 (1.7) | 11 (7) |  |
| Body mass index, mean (range) | 22.53 (15~36.3) | 23.01 (15.5~31.4) | 0.21 |
| Af, n (%) | 4 (3.3) | 5 (3.2) | 1 |
| DM, n (%) | 15 (12.5) | 21 (13.6) | 0.86 |
| History of cerebral infarction, n (%) | 9 (7.5) | 17 (11) | 0.41 |
| History of cancer within 5 years, n(%) | 22 (18.3) | 22 (14.3) | 0.41 |
| Anticoagulant therapy, n (%) | 8 (6.7) | 10 (6.5) | 1 |
| Charlson comorbidities 0 or 1, n (%) | 91 (76) | 118 (76.6) | 0.89 |
| 2-, n (%) | 29 (24) | 36 (23.3) |  |
| CEA ≤ 5, n (%) | 66 (55) | 102 (66) | 0.25 |
| > 5, n (%) | 32 (27) | 35 (23) |  |
| unknown, n (%) | 22 (18) | 17 (11) |  |
| **Operation time (min),  median (range)** | **118 (65~233)** | **203 (74~489)** | **<0.001** |
| **Bleeding amount (mL),  median (range)** | **27.5 (0~1506)** | **75.5 (0~4167)** | **0.009** |
| **PV length (mm), mean (range)** | **15.1 (9.9-20.4)** | **18.6 (13.4-22.9)** | **<0.001*** |
| Postoperative atrial filiation, n (%) | 2 (1.7) | 5 (3.2) | 0.47 |
| Postoperative cerebral infarction, n (%) | 1 (0.8) | 2 (1.3) | 1 |
| Postoperative complications, n (%) | 19 (16) | 33 (21.4) | 0.28 |
| **Postoperative stay (Day),  mean (range)** | **11 (4~55)** | **14.5 (3~56)** | **<0.001*** |
| Clinical stage I, n (%) | 105 (87.5) | 130 (84.4) | 0.64 |
| II, n (%) | 13 (10.8) | 19 (12.3) |  |
| III, n (%) | 2 (1.7) | 5 (3.2) |  |
| Pathological stage I, n (%) | 89 (74) | 102 (66) | 0.38 |
| II, n (%) | 18 (15) | 29 (19) |  |
| III, n (%) | 13 (11) | 23 (15) |  |
| Histology Adenocarcinoma, n (%) | 85 (71) | 115 (75) | 0.64 |
| Squamous cell carcinoma, n (%) | 25 (21) | 25 (16) |  |
| Others, n (%) | 10 | 14 (9) |  |

VC = vital capacity, FEV1.0% = percentage of forced expiratory volume in one second, Af = atrial fibrillation, DM = diabetes mellitus, CEA = carcinoembryonic antigen, RUL = right upper lobectomy, RML = right middle lobectomy, RLL = right lower lobectomy, LUL = left upper lobectomy, LLL = left lower lobectomy, CI = confidence interval, PV-last = procedure of dissecting pulmonary vein last, non-PV-last = a procedure of not dissecting pulmonary vein last.
